# Comparison of Four Molecular *In Vitro* Diagnostic Assays for the Detection of SARS-CoV-2 in Nasopharyngeal Specimens

**DOI:** 10.1128/JCM.00743-20

**Published:** 2020-07-23

**Authors:** Wei Zhen, Ryhana Manji, Elizabeth Smith, Gregory J. Berry

**Affiliations:** a Infectious Disease Diagnostics, Northwell Health Laboratories, Lake Success, New York, USA; b Department of Pathology and Laboratory Medicine, The Donald and Barbara Zucker School of Medicine at Hofstra/Northwell, East Garden City, New York, USA; Boston Children's Hospital

**Keywords:** COVID-19, EUA, molecular diagnostics, nasopharyngeal, real-time RT-PCR, SARS-CoV-2

## Abstract

Severe acute respiratory syndrome coronavirus 2 (SARS-CoV-2), the novel human coronavirus that causes coronavirus disease 2019 (COVID-19), was first discovered in December 2019 as the cause of an outbreak of pneumonia in the city of Wuhan, Hubei province, China. The clinical presentation of COVID-19 is fairly nonspecific, and symptoms overlap those of other seasonal respiratory infections concurrently circulating in the population. Furthermore, it is estimated that up to 80% of infected individuals experience mild symptoms or are asymptomatic, confounding efforts to reliably diagnose COVID-19 empirically. To support infection control measures, there is an urgent need for rapid and accurate molecular diagnostics to identify COVID-19-positive patients. In the present study, we evaluated the analytical sensitivity and clinical performance of the following four SARS-CoV-2 molecular diagnostic assays granted emergency use authorization by the FDA using nasopharyngeal swabs from symptomatic patients: the New York SARS-CoV-2 Real-time Reverse Transcriptase (RT)-PCR Diagnostic Panel (modified CDC) assay, the Simplexa COVID-19 Direct (Diasorin Molecular) assay, GenMark ePlex SARS-CoV-2 (GenMark) assay, and the Hologic Panther Fusion SARS-CoV-2 (Hologic) assay. This information is crucial for both laboratories and clinical teams as decisions on which testing platform to implement are made.

## Abstract (precis)

Severe acute respiratory syndrome coronavirus 2 (SARS-CoV-2), the novel human coronavirus that causes coronavirus disease 2019 (COVID-19), was first discovered in December 2019 as the cause of an outbreak of pneumonia in the city of Wuhan, Hubei province, China. The clinical presentation of COVID-19 is fairly nonspecific, and symptoms overlap those of other seasonal respiratory infections concurrently circulating in the population. Furthermore, it is estimated that up to 80% of infected individuals experience mild symptoms or are asymptomatic, confounding efforts to reliably diagnose COVID-19 empirically.

## INTRODUCTION

Severe acute respiratory syndrome coronavirus 2 (SARS-CoV-2) was identified as the causative agent for an outbreak of viral pneumonia that began in Wuhan, China, at the end of 2019 (1). On 11 March 2020, the classification of the SARS-CoV-2 epidemic was escalated to the level of a global pandemic by the World Health Organization (WHO) (1). The WHO has named the illness caused by SARS-CoV-2 “coronavirus disease 2019” (COVID-19). COVID-19 has since continued to spread across the globe, and as of 23 April 2020, over 2.65 million cases have been confirmed in more than 200 countries and territories, causing over ∼185,000 deaths. At the time of writing, more than ∼843,000 confirmed COVID-19 cases and ∼46,000 deaths had been reported in the United States according to the Centers for Disease Control and Prevention (CDC) and the database of the Center for System Science and Engineering (CSSE) at Johns Hopkins University (2, 3).

SARS-CoV-2 is the seventh coronavirus known to infect humans; SARS-CoV, Middle East respiratory syndrome-CoV (MERS-CoV), and SARS-CoV-2 can cause severe disease, whereas seasonal coronavirus HKU1, NL63, OC43, and 229E are associated with mild symptoms (4). Coronaviruses are members of a diverse family of large RNA viruses that are known to be involved in zoonotic transmission between a wide variety of animals and humans. Coronaviruses generally target epithelial cells in the respiratory and gastrointestinal tracts, and viral shedding can occur from these sites. Infection caused by coronaviruses can therefore typically be transmitted through several different routes, including the aerosol and fecal-to-oral routes, with fomites often playing an important role in the infection cycle (5). Notably, coronaviruses possess a distinctive morphological feature, consisting of a ring of spike proteins on the outer surface of the virus, giving the appearance of a halo or corona. In addition to inspiring the name of the coronavirus genus, the spike proteins are also essential for infection of host cells. The SARS-CoV-2 spike protein recognizes and binds to human cellular receptor angiotensin-converting enzyme 2 (ACE2) and subsequently mediates fusion of the viral and host cell membranes, allowing the virus to gain entry (6, 7). The ACE2 receptor is found on epithelial cells of the lungs and small intestines (8).

Infection with SARS-CoV-2 can cause mild to severe respiratory illness, and symptoms include fever, cough, and shortness of breath. However, some populations experience severe, rapidly progressive, and fulminant disease. Among these populations are older adults and people who have serious underlying medical conditions (e.g., heart disease, diabetes, lung disease, and immunosuppression) (9). Unfortunately, many elements, some intrinsic to the virus and others seasonal, have lessened the effectiveness of traditional infection control measures. The combination of high rates of human-to-human transmission (*R*_0_ = 2.0 to 2.5), stability of the virus in aerosols and on surfaces, the fairly nonspecific clinical presentation of COVID-19, and coincidence of the onset of COVID-19 with the active season of other respiratory viruses (e.g., influenza virus and respiratory syncytial virus [RSV]) in many parts of the world presents a major challenge to efforts to stop the pandemic from spiraling into a more severe global health emergency (9, 10).

During the early stages of the epidemic, both national and international agencies rushed to initiate the process of mass production of test reagents and issued an emergency use authorization (EUA) for the U.S. CDC COVID-19 real-time reverse transcriptase (RT)-PCR assay (11). Despite the collective efforts, laboratories are still facing reagent supply shortages, lack of instrument access, an inability to perform high-complexity testing, and increased staffing needs, leaving a gap in the ability of health care providers to rapidly diagnose and manage patients. The need to implement a sensitive, accessible, and rapid diagnostic test for the detection of COVID-19 is clear. In this study, we evaluated the analytical and clinical performance of four SARS-CoV-2 molecular diagnostic assays granted EUA by the FDA, including the modified CDC, DiaSorin Molecular, GenMark, and Hologic assays. These assays are authorized for the qualitative detection of SARS-CoV-2 in clinical specimens obtained from symptomatic patients and were evaluated using nasopharyngeal swab specimens.

## MATERIALS AND METHODS

### Specimen collection and storage.

Sterile nylon, Dacron, or rayon swabs with flexible plastic shafts were used to collect nasopharyngeal specimens (NPS) from symptomatic patients. After collection, swabs were placed into 3 ml of sterile universal transport medium (UTM; various manufacturers). Specimens were transported and tested as soon as possible after collection. Before testing, samples were subjected to vortex mixing for 3 to 5 s and a calibrated pipette was used to transfer the specimen volume specified by each manufacturer’s instructions for use. The samples were kept for up to 72 h at 2 to 8°C after collection before initial testing. Following routine testing, samples were aliquoted and stored in a freezer at −80°C until comparator testing.

### The New York SARS-CoV-2 Real-time Reverse Transcriptase (RT)-PCR Diagnostic EUA Panel (modified CDC assay).

This assay is a modified version of the CDC 2019-Novel Coronavirus (2019-nCoV) Real-Time RT-PCR Diagnostic EUA Panel validated by the Wadsworth Center (Albany, NY) using the same primer and probe sets as the CDC assay for nucleocapsid gene 1 (N1) and N2 targets and the human RNase P gene (RP) but excluding the N3 primer and probe set. A 110-μl volume of patient specimen was extracted by the use of a NucliSENS easyMAG platform (bioMérieux, Durham, NC) according to manufacturer’s instructions, with a nucleic acid elution volume of 110 μl. For each specimen, three Master Mix sets that included N1, N2, and RNase P were prepared, and 15 μl of each master mix was dispensed into appropriate wells, followed by 5 μl of extracted sample. Each run also included a no-template control (NTC), a negative extraction control, and a SARS-CoV-2-positive control. Amplification was performed on an Applied Biosystems 7500 Fast Dx real-time PCR system (Thermo Fisher Scientific, Waltham, MA). The results interpretation algorithm for reporting a positive specimen requires both N1 and N2 targets to be detected.

### Simplexa COVID-19 Direct EUA (Diasorin Molecular LLC, Cypress, CA).

Testing with the DiaSorin Molecular Simplexa COVID-19 Direct EUA assay was performed according to the manufacturer’s instructions for use. A 50-μl volume of Simplexa COVID-19 Direct kit reaction mix (MOL4150) was added to the “R” well of the 8-well direct amplification disc (DAD) followed by addition of 50 μl of nonextracted nasopharyngeal swab (NPS) sample to the “SAMPLE” well. Data collection and analysis were performed with LIAISON MDX Studio software. The assay targets two different regions of the SARS-CoV-2 genome, including the surface (S) gene and open reading frame 1ab (ORF1ab), differentiated with 6-carboxyfluorescein (FAM) and JOE fluorescent probes. An RNA internal control (Q670 probe) is used to detect RT-PCR failure and/or inhibition. The results interpretation algorithm for reporting a positive specimen requires only one of the two targets to be detected (S or ORF1ab gene).

### GenMark ePlex SARS-CoV-2 assay EUA (GenMark Diagnostics, Carlsbad, CA).

Testing with the ePlex SARS-CoV-2 was performed according to the manufacturer’s instructions for use. Briefly, after vortex mixing was performed for 3 to 5 s, 200 μl of the primary NPS sample was aspirated into the sample delivery device (SDD) provided with the ePlex SARS-CoV-2 panel kit and subjected to vortex mixing once again for 10 s. The entire volume of the SDD was dispensed into the sample loading port of the SARS-CoV-2 test cartridge, followed by firmly pushing down the cap to securely seal the sample delivery port. Each cartridge was bar-coded and scanned with the ePlex instrument and inserted into an available bay. Upon the completion of the assay run, the ePlex SARS-CoV-2 report was generated. The GenMark ePlex SARS-CoV-2 assay amplifies and detects the SARS-CoV-2 virus nucleocapsid (N) gene.

### Hologic Panther Fusion SARS-CoV-2 EUA (Hologic Inc., San Diego, CA).

The Fusion SARS-CoV-2 assay was performed according to the manufacturer’s instructions for use. A 500-μl volume of NPS was lysed by transfer to a specimen lysis tube containing 710 μl lysis buffer. The input volume per sample for extraction is 360 μl. Internal control S (IC-S) was added to each test specimen and to the controls via working Panther Fusion capture reagent S (wFCR-S). Hybridized nucleic acid was then separated from the specimen in a magnetic field. After wash steps, 50 μl of purified RNA was eluted. Then 5 μl of eluted nucleic acid was transferred to a Panther Fusion reaction tube already containing oil and reconstituted master mix. The Panther Fusion SARS-CoV-2 assay amplifies and detects two conserved regions of the ORF1ab gene in the same fluorescence channel. The two regions are not differentiated, and amplification of either or both regions results in a fluorescent ROX signal. The results interpretation algorithm for reporting a positive specimen requires only one of the two targets to be detected (the ORF1a or ORF1b gene).

### Analytical sensitivity.

Limit of detection (LoD) determinations were performed using a SARS-CoV-2 synthetic RNA quantified control (SARS-CoV-2 Standard) containing five gene targets (E, N, ORF1ab, RNA-dependent RNA polymerase [RdRP], and S genes of SARS-CoV-2) from Exact Diagnostics (catalog no. COV019; Exact Diagnostics, Fort Worth, TX). A starting concentration of 200,000 copies/ml control was used to generate a dilution panel. The control was diluted in Ambion RNA storage solution (catalog no. AM7001; Thermo Fisher Scientific, Waltham, MA) and aliquoted for testing in order to obtain a maximum of 12 replicates at the following concentrations: 20,000, 2,000, 1,000, 500, 100, 50, and 5 copies/ml. The LoD was determined by two methods: positive-rate analysis and Probit analysis. A positive rate was defined as the lowest dilution at which all replicates gave positive test results with a 100% detection rate. The LoD by Probit was defined as the lowest detectable dilution at which the synthetic RNA quantified control (measured in numbers of copies per milliliter) gave a positive result with a 95% probability of detection. The final LoD was based on results of Probit analyses and on each manufacturer’s claimed results interpretation algorithm, which determines whether a specimen result is positive, negative, or inconclusive on the basis of the gene targets detected.

### Study design.

A total of 104 nasopharyngeal specimens (88 retrospective and 16 prospective) originally submitted during March and April of 2020 to Northwell Health Laboratories for routine COVID-19 testing on the GenMark were selected for this study. Of the 104 specimens analyzed, 51 of the samples were positive and 53 were negative. Retrospective frozen samples were thawed, pipetted into separate aliquots, and immediately tested by the modified CDC, DiaSorin Molecular, and Hologic assays in parallel. Prospective specimens were performed fresh on each platform in parallel at the time of patient testing. The study population included patients of all ages and both genders presenting with signs and/or symptoms of COVID-19 infection. Specimens selected for this study included positive specimens spanning the range of positivity and also specimens with low viral loads (characterized by high cycle threshold [*C_T_*] values). In addition, specimens were selected to represent the true positivity rate determined by us at the time this study was performed (50% to 60%). The manufacturer’s specifications are summarized in Table 1.

**TABLE 1:** Overview of four molecular *in vitro* diagnostic EUA assays used in this study^*a*^

Assay	Sample type	Sample vol required (μl)	Extraction required	Detection platform/system	Target region(s) of SARS-CoV-2	Analytical sensitivity per claim
Modified CDC	NPS, OPS, sputum	110	Yes	ABI 7500 Fast Dx	N (N1 and N2)	500 copies/ml
Diasorin Molecular	NPS, NS, BAL fluid	50	No	LIAISON MDX	S and ORF1ab	500 copies/ml
GenMark	NPS	200	Yes (automated)	ePlex	N	100,000 copies/ml
Hologic	NPS, OPS	500	Yes (automated)	Panther Fusion	ORF1ab	1 × 10^−2^ TCID_50_/ml

a BAL, bronchoalveolar lavage; NPS, nasopharyngeal swabs; NS, nasal swabs; OPS, oropharyngeal swabs; TCID_50_, 50% tissue culture infective dose.

### Discordant analysis.

Results were considered discordant when one molecular assay did not agree qualitatively (detected or not detected) with the other three assay results. In such cases, molecular testing was repeated for the discordant assay.

### Workflow evaluation.

Workflow was evaluated by the use of a stopwatch to measure the amount of time needed for each step being evaluated, including hands-on time (HoT), assay run time, and total turnaround time (TAT). HoT, assay run time, and TAT were calculated using the throughput of samples per run.

### Statistical methods.

The reference standard was established as a “consensus result” which was defined as the result obtained by at least three of the four molecular diagnostic assays. Positive percent agreement (PPA), negative percent agreement (NPA), positivity rate, Kappa (κ), Probit, and two-sided (upper/lower) 95% confidence interval (CI) data were calculated using Microsoft Office Excel 365 MSO software (Microsoft, Redmond, WA). Cohen’s kappa values (κ) were also calculated as a measure of overall agreement, with values representing levels of agreement categorized as follows: almost perfect (>0.90), strong (0.80 to 0.90), moderate (0.60 to 0.79), weak (0.40 to 0.59), minimal (0.21 to 0.39), or none (0 to 0.20) (12, 13). Probit analyses were used for the determination of the analytical sensitivity of the studies expressed in numbers of copies per milliliter. The dose-response 95th percentile (with 95% confidence interval [CI]) model was assessed using the Finney and Stevens calculations (14).

## RESULTS

### Analytical sensitivity.

A serial dilution of SARS-CoV-2 control panel was tested to determine the LoD, defined as the minimum concentration with detection values of 100% by positive rate and 95% by Probit analysis. The LoD established by percent positive rate ranged from 1,000 copies/ml by both the GenMark and the modified CDC assays to 50 copies/ml by the DiaSorin Molecular assay (Table 2). The LoD results were further subjected to Probit analysis. The 95% detection limit values for the CDC assay were 779 ± 27 copies/ml for the N1 gene and 356 ± 20 copies/ml for the N2 gene. For the DiaSorin Molecular assay, the 95% detection limit values were 39 ± 23 copies/ml for the S gene and 602 ± 28 copies/ml for ORF1ab. For the Hologic assay, the 95% detection limit value was 83 ± 36 copies/ml for ORF1ab. Probit analysis could not be performed for the GenMark assay (Table 2). The final LoD values, according to the assay results interpretation algorithm from each manufacturer, ranged from 1,000 copies/ml by the GenMark assay to 39 ± 23 copies/ml by the DiaSorin Molecular assay (Table 2).

**TABLE 2:** Summary of limit of detection results

Molecular assay	Target region	No. (%) of detected replicates at indicated dilution (copies/ml)^*a*^						No. of copies/ml (95% CI)^*b*^	
		2,000	1,000	500	100	50	5	Probit	Final LoD^*c*^
Modified CDC	N1	4/4 (100)	**8/8 (100)**	7/10 (70)	1/10 (10)	0/8 (0)	1/4 (25)	779 ± 27	779 ± 27
	N2	4/4 (100)	8/8 (100)	**10/10 (100)**	6/10 (60)	3/8 (38)	0/4 (0)	356 ± 20	

DiaSorin Molecular	S	1/1 (100)	10/10 (100)	10/10 (100)	10/10 (100)	**8/8 (100)**	0/4 (0)	39 ± 23	39 ± 23
	ORF1ab	1/1 (100)	**10/10 (100)**	8/10 (80)	4/10 (40)	1/8 (13)	0/4 (0)	602 ± 28	

GenMark	N	10/10 (100)	**9/9 (100)**	7/10 (70)	1/10 (10)	1/4 (25)	0/4 (0)	NA^*d*^	1,000

Hologic	ORF1ab	3/3 (100)	9/9 (100)	12/12 (100)	**12/12 (100)**	5/9 (56)	0/6 (0)	83 ± 36	83 ± 36

a The limit of detection by positive rate for each assay is highlighted in bold.

b 95% CI, upper/lower (±) 95% confidence interval.

c The final LoD data were determined on the basis of each manufacturer’s results interpretation algorithm.

d NA, not applicable.

### Clinical performance of four EUA SARS-CoV-2 (COVID-19) molecular assays.

Following testing of 104 clinical specimens, the modified CDC, DiaSorin Molecular, and Hologic EUA molecular assays demonstrated a PPA of 100% (51/51), while the GenMark ePlex SARS-CoV-2 EUA panel showed a PPA of 96% (49/51). A NPA of 100% (53/53) was observed for GenMark and DiaSorin Molecular, while the NPA values ranged from 98% (52/53) for CDC to 96% (51/53) for Hologic (Table 3).

**TABLE 3:** Clinical performance comparison of four EUA molecular assays for the detection of SARS-CoV-2 in nasopharyngeal swab specimens (*n* = 104)

Molecular assay and result	No. of results by reference standard^*a*^		Kappa (κ) (± 95% CI)^*b*^^,^^*c*^	PPA (± 95% CI)^*c*^	NPA (± 95% CI)^*c*^
	Positive	Negative			
Modified CDC					
Positive	51	1^*d*^	0.98 (0.94–1)	100 (0.93–1)	98 (0.89–0.99)
Negative	0	52			

DiaSorin Molecular					
Positive	51	0	1.0 (0.99–1)	100 (0.93–1)	100 (0.93–1)
Negative	0	53			

GenMark					
Positive	49	0	0.96 (0.91–1)	96 (0.87–0.99)	100 (0.93–1)
Negative	2^*e*^	53			

Hologic					
Positive	51	2^*f*^	0.96 (0.91–1)	100 (0.93–1)	96 (0.87–0.99)
Negative	0	51			

a The reference standard was defined as the result obtained from at least 3 of the 4 molecular assays.

b Kappa values representing levels of agreement are categorized as follows: >0.90, almost perfect; 0.80 to 0.90, strong; 0.60 to 0.79, moderate; 0.40 to 0.59, weak; 0.21 to 0.39, minimal; (0 to 0.20), none.

c ± 95% CI, upper/lower 95% confidence interval.

d This one sample had cycle threshold (*C_T_*) values of 38.9 and 39.6 for N1 and N2, respectively, by the CDC assay.

e Cycle threshold (*C_T_*) value undetermined.

f These two samples had cycle threshold (*C_T_*) values of 36.2 and 38.5 by the Hologic assay.

Details of discordant sample analysis results are shown in Table 4. A total of five discordant samples were found among three of the four platforms. One false-positive sample (sample A) had *C_T_* values of 38.9 and 39.6 the for N1 and N2 genes, respectively, on initial testing by the modified CDC assay. After repeating extraction and retesting, the sample was determined to be negative. Two samples (sample B and sample C) were considered false negative by GenMark but positive by the other three methods. After reprocessing and retesting, the GenMark assay was able to detect both samples as positive. Two additional false-positive samples (sample D and sample E) were found by the Hologic assay; the original samples were retested and were found to be positive and negative, respectively. Following retesting of the five discordant samples, the GenMark ePlex SARS-CoV-2 EUA panel showed an improvement of the PPA to 100% (51/51). Additionally, 100% NPA (53/53) was obtained for the CDC assay, while Hologic improved to 98% (52/53) (Table 4).

**TABLE 4:** Details of discordant sample analysis

Sample ID	Reference standard result	SARS-CoV-2 molecular assay result (*C_T_*)^*a*^				
		Modified CDC (N1/N2)	DiaSorin Molecular (S/ORF1ab)	GenMark	Hologic	Comment
A	NEG	**POS** (38.9/39.6)	NEG	NEG	NEG	Sample assay was repeated by CDC and determined NEG
B	POS	POS (35.5/ 34.5)	POS (31.9/31.8)	**NEG**	POS (35.0)	Sample assay was repeated by GenMark and determined POS
C	POS	POS (35.3/35.0)	POS (29.3/29.9)	**NEG**	POS (33.0)	Sample assay was repeated by GenMark and determined POS
D	NEG	NEG	NEG	NEG	**POS** (36.2)	Sample assay was repeated by Hologic and determined POS
E	NEG	NEG	NEG	NEG	**POS** (38.5)	Sample assay was repeated by Hologic and determined NEG

a Discordant sample results are highlighted in bold. CT, cycle threshold; ID, identifier; NEG, negative; POS, positive.

### Workflow evaluation.

HoT, run time, and overall TAT to results were assessed for all preanalytical, analytical, and postanalytical steps for all four platforms. Results of the workflow assessment are shown in Table 5. The longest HoT was the Hologic assay at ∼2 h followed by the modified CDC assay at ∼1 h 30 min. Very comparable HoT results were found for DiaSorin Molecular and GenMark with times of 16 min and 12 min, respectively. The run time averaged 90 min for modified CDC, DiaSorin Molecular, and GenMark. Hologic was the exception, with 4 h 35 min of run time (Table 5). Overall TAT assessment, from sample to results, showed DiaSorin Molecular with the lowest TAT to results, followed by the GenMark, modified CDC, and Hologic assays with the highest overall times (Table 5).

**TABLE 5:** Throughput and workflow evaluation for four EUA molecular SARS-CoV-2 assays

SARS-CoV-2 molecular assay	Throughput (no. of samples per run)	Input vol per sample/ elution vol	HoT (per run)	Assay run time	User results interpretation	Overall TAT
Modified CDC	24	110 μl/110 μl	∼1.5 h	∼90 min	Yes	∼3.0 h
DiaSorin Molecular	8 per disc	50 μl/NA^*a*^	<16 min	∼90 min	No	∼1.8 h
GenMark	6 per tower	200 μl/NA	<12 min	∼90 min	No	∼1.7 h
Hologic	120	360 μl/50 μl	∼2.0 h	∼4 h 35 min	No	∼6.6 h

a NA, not applicable.

## DISCUSSION

In this study, we compared four different platforms for the detection of SARS-CoV-2 in patient specimens collected during March and April of the 2020 COVID-19 outbreak in the United States. We were able to make several observations, including LoD, overall workflow comparisons, and how each test performed in a head-to-head clinical comparison. Accurate and actionable results have been at the core of medical decision-making during this current outbreak, both in the inpatient and the outpatient settings. For hospitalized patients, results are critical for clinical management as well as for infection control and cohorting for bed management. Likewise, results are just as critical in the outpatient setting as the basis for social distancing measures to slow the spread of infection. To that end, false-negative results are particularly troubling, since they inevitably lead to more exposures. TAT is also critical for allocation of limited resources, such as the limited availability of isolation rooms and real-time cohorting decisions. In addition, health care workers need rapid results to ensure that they are not exposing the patients whom they are treating. Moreover, levels of personal protective equipment (PPE) required by health care professionals also vary depending on whether a patient is COVID-19 positive, requiring a short TAT to preserve precious resources. Considering the transmissibility of SARS-CoV-2, which has recently been estimated to have a basic reproduction number (*R*_0_) of 2.2, meaning that on average, each infected person can spread the infection to an additional two persons, a false-negative result can be devastating (15, 16). This is especially true in vulnerable patient populations such as the elderly (especially people living in a nursing home or long-term-care facility), the immunocompromised, and people with preexisting medical conditions (17, 18).

Our data suggest that all four PCR methods yielded comparable results (κ ≥ 0.96); however, we did observe a notable difference in the PPA of the methods during this large-scale evaluation of EUA *in vitro* diagnostic assays. Our study showed that the DiaSorin Molecular and Hologic Fusion assays outperformed both the modified CDC and GenMark assays with respect to overall LoD, with GenMark having the overall highest LoD of all four platforms evaluated. DiaSorin Molecular had the lowest LoD (39 ± 23 copies/ml), closely followed by Hologic (83 ± 36 copies/ml). The modified CDC assay showed a final LoD of 779 ± 27 copies/ml based on the results interpretation algorithm. It is worth mentioning that this assay requires both targets to be fully detected; thus, clinical samples falling in this concentration range would be identified and testing repeated, potentially requiring additional TAT and laboratory labor. In contrast, GenMark was able to detect 100% of the replicates only at 1,000 copies/ml and was not able to reliably detect replicates below 1,000 copies/ml; thus, patient specimens below this concentration range could potentially be missed. One important limitation to mention is that sensitivity using Probit analysis could not be calculated for GenMark since *C_T_* values are not available as part of the ePlex system result interpretation.

The clinical correlation was also consistent with the LoD findings, where both the DiaSorin Molecular and Hologic assays had 100% PPA and detected all specimens deemed positive by the consensus standard (interpretation of three of four evaluated assays as the “gold standard”), whereas GenMark missed two positive specimens (which were subsequently detected by GenMark upon repeat testing). DiaSorin Molecular and GenMark showed 100% NPA, while the Hologic and CDC assays initially had two and one discordant results, respectively. Repeat testing of these three specimens showed that for Hologic, sample D repeated as positive a second time and was therefore potentially a false positive and sample E was negative upon repeat testing, meaning this result could have previously been a false positive as well. The continued discordant result from sample D could potentially be attributed to specificity issues, since DiaSorin Molecular exhibited a slightly lower LoD. Repeat testing of sample A on the modified CDC assay was negative. Considering the LoD of the modified CDC assay, coupled with the fact that both DiaSorin Molecular and Hologic gave negative test results for sample A, this was likely a false-positive result. While all 4 assays could reliably produce results for most patient specimens in our study, GenMark lacked sensitivity, initially missing two low-level-positive specimens, and this could easily have impacted patient diagnosis by missing true-positive patient results.

With respect to the HoT and TAT of the four assays in this study, the throughput and workflow evaluation results are clearly shown in Table 5 and are based on lab technologist experience in our laboratory. As a routine real-time RT-PCR assay, the modified CDC assay requires nucleic acid extraction, master mix preparation and PCR setup, and standard PCR amplification, as well as interpretation of the results. This involves several manual steps, needing about 1 h 30 min HoT and an approximate overall TAT of 3 h for processing of 24 specimens. The DiaSorin Molecular and GenMark assays have comparatively similar HoT and TAT, based on processing 8 samples per disc on the DiaSorin LIAISON MDX and 6 cartridges per tower in the GenMark ePlex. Clinical laboratories may decide to purchase additional instruments to allow testing of more samples at a time in order to satisfy patient testing volume requirements. The Hologic Panther Fusion platform has more of an automated workflow, with five samples processed at a time after loading. The sample-to-answer time for the first five samples is 2 h 40 min, followed by 5 results every 5 min after loading 120 samples; the total assay run time for 120 specimens is approximately 4 h 35 min. Note that the Hologic platform has longer HoT, because the technologist has to load the primers, probes, and other consumables and because 120 clinical samples have to be manually transferred to sample lysis buffer tubes. These steps, especially the pipetting of the specimen into the lysis tube, can be somewhat labor-intensive and time-consuming, bumping the overall TAT for 120 specimens closer to the 7-h mark. It is important to emphasize that each platform has its advantages. For workflow, TAT, and ease of use, the three sample-to-answer platforms (DiaSorin Molecular, Hologic, and GenMark) outperformed the modified CDC assay, which is a manual assay requiring many steps, specialized personnel, and separate areas for processing and performing the test. The Hologic platform is more appropriate for high-volume testing, while the DiaSorin Molecular and GenMark systems both work well in an environment where rapid results and low-to-moderate testing volumes are required.

This study had several limitations that should be mentioned. First, this was a single-center study, and the majority of the specimens were frozen after initial testing on the GenMark assay. While these limitations were present, they were minimized by the fact that the GenMark assay (which was the least sensitive platform in the analysis) actually had a potential competitive advantage, since it was the assay initially performed on fresh specimens. Second, while the number of specimens included in the clinical correlation was only 104, the patient samples spanned the entire range of clinical positives (including inclusion of specimens with low viral loads) and reflected our overall true positivity rate, which was between 50% and 60% during this time period of the COVID-19 outbreak.

In summary, we have evaluated four molecular *in vitro* diagnostic assays for the qualitative detection of SARS-CoV-2 in nasopharyngeal specimens. The data from our evaluation suggest that the modified CDC, DiaSorin Molecular, Hologic, and GenMark assays performed similarly (κ ≥ 0.96) and that all but the CDC assay can function in a sample-to-answer capacity. The GenMark assay, however, was less sensitive and had a higher LoD than both the DiaSorin Molecular and Hologic assays. Considering the design of all four assays, differences that could affect assay performance could include characteristics such as input volume of initial specimen, RNA purification and elution volume differences, and overall differences in gene targets. The DiaSorin Molecular platform has a lower testing volume capability than the Hologic assay (8 specimens/disc run versus 120 specimens loaded at once) but has a short TAT and requires less reagent/sample preparation. All of these parameters, along with patient care needs, may assist clinical laboratories to identify and choose the correct testing platform that best fits their needs for the diagnosis of patients infected with this novel human coronavirus.
